# Study the plasmonic property of gold nanorods highly above damage threshold via single-pulse spectral hole-burning experiments

**DOI:** 10.1038/s41598-021-01195-5

**Published:** 2021-11-15

**Authors:** Zibo Wang, Zhe Kan, Mengyan Shen

**Affiliations:** Department of Physics and Applied Physics, University of Massachusetts Lowell, 220 Pawtucket St, Lowell, MA 01854 USA

**Keywords:** Nanophotonics and plasmonics, Nonlinear optics, Nanoparticles

## Abstract

Intense femtosecond laser irradiation reshapes gold nanorods, resulting in a persistent hole in the optical absorption spectrum of the nanorods at the wavelength of the laser. Single-pulse hole-burning experiments were performed in a mixture of nanorods with a broad absorption around 800 nm with a 35-fs laser with 800 nm wavelength and 6 mJ/pulse. A significant increase in hole burning width at an average fluence of 10^6^ J/m^2^ has been found, suggesting a tripled damping coefficient of plasmon. This shows that the surface plasmonic effect still occurs at extremely high femtosecond laser fluences just before the nanorods are damaged and the remaining 10% plasmonic enhancement of light is at the fluence of 10^6^ J/m^2^, which is several orders of magnitude higher than the damage threshold of the gold nanorods. Plasmon–photon interactions may also cause an increase in the damping coefficient.

## Introduction

Gold nanorods attract our attention due to their applications in medicine, chemistry and physics, including biosensing^1^, drug delivery^2^, cellular imaging^3,4^, cancer therapy^5^, chemical analysis^6,7^, catalysts^8–12^, electronics^13,14^ and nonlinear processes^15^. These applications utilize the plasmonic effect: the interaction of conductive electrons in the metallic structures with electromagnetic fields^16^. The effects of the particles’ size, shape, and environment on plasmonic absorption have also been studied extensively^17,18^. In particular, gold nanoparticles are especially favored for their physical and chemical stability as well as the tunability of their plasmonic properties^19^.

The resonant plasmons lead to a nano-focusing effect, or electromagnetic (EM) enhancement, by coupling a resonating bulk optical radiation into the strongly localized near field^20,21^. The simplest expression that describes the EM enhancement for a small spherical particle can be written as:

1 $$\frac{{E_{local} }}{{E_{0} }} \propto \frac{1}{{\varepsilon (\omega ) + 2\varepsilon_{s} }}$$

where *E*_*local*_ is the local electric field due to the polarization of the particle, ω is the frequency of the incident light, ε(ω) and ε_s_ are the dielectric constants of the small metal including that of its surrounding at the frequency ω, and *E*_0_ is the electric field of the incident light. According to this equation, the local electric field will approach infinity when the dielectric constant approaches $- 2\varepsilon_{s}$, however the imaginary part of the dielectric constant keeps the EM enhancement finite. The light intensity enhancement factor $(\frac{{E_{local} }}{{E_{0} }})^{2}$ is approximately proportional to $\frac{1}{{\Gamma^{2} }}$, where $\Gamma$ is the damping coefficient to describe the electron scattering in the Drude model. The light intensity enhancement factor reaches the order of 10^4^^22^ which is due to weak light-nanoparticle interactions. Whether this enhancement factor is still large when the laser energy is high, can be investigated by studying the light intensity dependence of the homogeneous broadening of $\Gamma$, which can be directly obtained from the measurement of the confocal light scattering spectroscopic microscopy^23^ and a scanning near-field optical microscopy of single particles^24^. $\Gamma$ can also be obtained from the optical absorption and the size/shape distributions of an assembly of particles^24^. However, these methods do not provide instantaneous information of $\Gamma$ right before the time that the nanoparticles are melted or damaged when they interact with an intense femtosecond laser pulse.

The homogeneous broadening of $\Gamma$ of a gold nanoparticle is several tens of nm, which is much larger than that of semiconductor nanoparticles. When light irradiates a semiconductor nanoparticle, the particle may be photoionized^25^, which results in a photodarkening effect^26^, spectral diffusion^27,28^, and a persistent hole-burning phenomenon^29^. The hole-burning effect provides an effective method to study the homogeneous broadening of the optical spectrum of a semiconductor nanoparticle^29^.

Kudryashov et al*.* reported a broadening of the localized plasmonic resonance at about 520 nm of gold spheres that are 160-nm in diameter when irradiated by a 100-fs pulse laser at 800 nm with an average fluence of about 10^4^ J/m^2^^30^. This phenomenon was attributed to an increase in the free carrier concentration in s-band, which is induced by strong excitation and electron heating^30^. Here, we report the study of resonantly exciting the longitudinal plasmon of gold nanorods at 800 nm. When an intense laser pulse irradiates Au nanorods, the nanorods absorb the light through the resonant-plasmon excitation or weak non-resonant excitation. The absorbed energy heats up the nanorods, and the rods may be deformed or damaged^31^. This may result in a persistent hole in the absorption spectra. The hole width is due to the homogeneous broadening $\Gamma$ of the resonant plasmon excitation because the non-resonant excitation cannot generate a hole but a broader spectrum shift. The inhomogeneous absorption band of an assembly of gold nanoparticles is generally a little broader than homogeneous broadening nanoparticles^24^. We have mixed gold nanorods with different sizes, similarly to DeSantis et al.^32^ to increase the inhomogeneous absorption band, but we used a single-pulse design to eradicate influences from previous laser pulse irradiations. The damping factor of nanorods can thus be studied explicitly under a simplified process of the single pulse-irradiation. Here, we report a laser intensity dependence of the $\Gamma$ obtained with this single pulse hole burning method.

A significant increase in hole burning width from 80 to 250 nm at 10^6^ J/m^2^ fluences has been found, suggesting a tripled damping coefficient of plasmon. This fluence is 10^4^ times higher than the threshold for gold nanorods to be completely damaged, which is experimentally estimated to be 100 J/m^2^ with femtosecond pulses^33^. The increase in hole burning width shows that the surface plasmonic effect holds at extreme femtosecond laser fluences, achieving 10% plasmonic enhancement of light at the fluence of about 10^6^ J/m^2^. The results suggest that an appropriate gold nanostructure can still be used to enhance or nano-focus an intensive femtosecond laser pulse. For example, potential applications are for triggering nuclear reactions^34^ and for generating coherent high-energy particle beams. By using a plasmon–photon interaction^35^, we understand the increase of the damping coefficient because the boson property of photons cannot be neglected when the photon number or light intensity is high.

## Experimental

**Figure 1:**
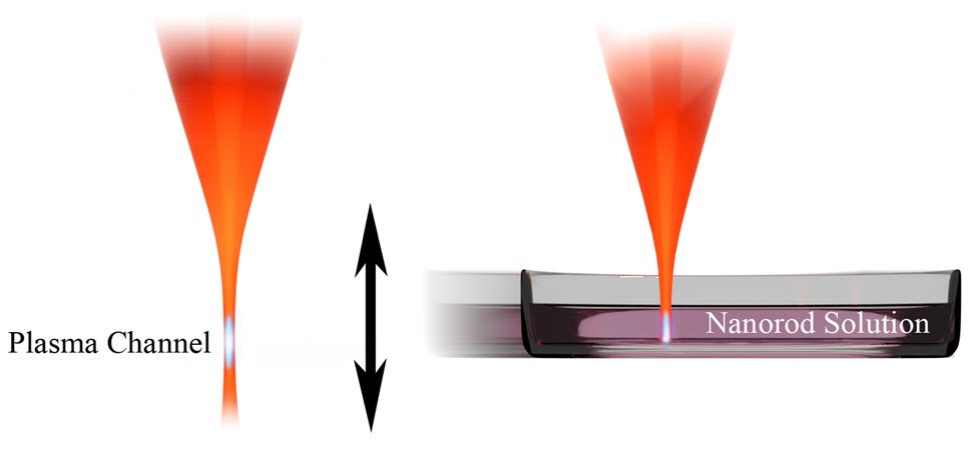
An illustration of the experimental setup for the femtosecond laser irradiation, where the laser is focused vertically as shown in orange. The drawing on the left shows the plasma channel (in bright blue) formed in the air and the drawing on the right shows the surface of the nanorod solution is placed above the air plasma channel position. The cuvette can move vertically with a translation stage to vary the laser spot size on the surface of the solution.

We used gold nanorods made by Nanopartz INC which are 10 nm in diameter and their length ranges from 20 to 50 nm. The nanorods are dissolved in water and citrate is used as the ligand. The concentration is 2 × 10^12^ rod per ml before dilution. A femtosecond laser of 800 nm in wavelength, repetition rate of 1 kHz, pulse duration of 35 fs, and pulse energy of 6 mJ were used to excite the gold nanorods. To prevent the 35-fs laser pulse from stretching, the horizontal laser beam was directly tightly focused 200 mm below the beam with an off-axis parabolic gold-coated mirror. The focal point of the mirror was placed below the top surface of the sample nanorod solution with a depth of about 3 mm. A translation stage carrying the solution in a glass petri dish is moved vertically to adjust the spot size upon contact, varying the fluence from 300 to 10^6^ J/m^2^ as shown in Fig. 1. A variable neutral-density filter was also used to vary the energy of each pulse so the fluence ranges from 10 to 300 J/m^2^. An air plasma channel (or filament) will form around the focal point if the sample is removed as illustrated in Fig. 1. The length of the air plasma channel was observed to be about 2.5 mm. For the maximum fluence excitation, the focal point of the mirror was placed about 2 mm below the front surface of the sample nanorod solution, so that the laser fluence is lower than the plasma channel formation threshold in the air before the light enters the nanoparticle solution^36^ as shown in Fig. 1. The spot size of the laser just before the light entering the solution is estimated to be about 50 μm (which is also the size of the cross-section of the following filament in the solution) for calculating the maximum fluence. The minimum spot size of laser damage on a silicon surface in water is measured to be about 50 μm, which agrees with the estimation^37–39^.

After the light enters the solution, its energy is not uniformly distributed in the plasma channel because of the self-focusing and filament formation. The average separation between nanorods in the solution is estimated to be 3 μm, the size of the optical cross-section of a nanorod is more than a hundred of nanometers which is several times larger than its geometry size^40,41^. The laser energy delivered into the nanorod may be much larger than the direct estimation with the laser fluence and its geometry size, which needs further study. Therefore, the fluence obtained with the laser pulse energy and the laser spot size is an average fluence. i.e., the fluences for the following experiments are of average fluences.

When the laser propagates in the solution for a distance of about 3 mm, white light is generated^30^. The white light with a broad spectrum may uniformly excite and deform all the nanorods with different sizes^19^, which cannot generate a hole but a broad spectrum shift or change. Thus, the white light does not contribute to the width of the spectral hole formed with the resonant excitation at 800 nm in this work.

During the experiment, an optical chopper wheel chopped the pulse train into 50 Hz and another electric shutter further reduced the repetition rate to 2 Hz. Irradiation time was actively controlled by a programmable shutter, and a translation stage moves the sample at about 1 mm/s horizontally as shown in Fig. 1. This setup gives enough time for the sample to move to a new position for single pulse irradiation, since nanoparticles move slowly in a liquid^42^. Single-pulse irradiation scanning prevents most of the nanorods from being damaged by multiple pulses and decreases the chance of the nanorods from being reshaped or damaged from further irradiation. This scanning technique makes the measurement possible for the homogeneous broadening of a nanorod’s plasmon resonance.

**Figure 2:**
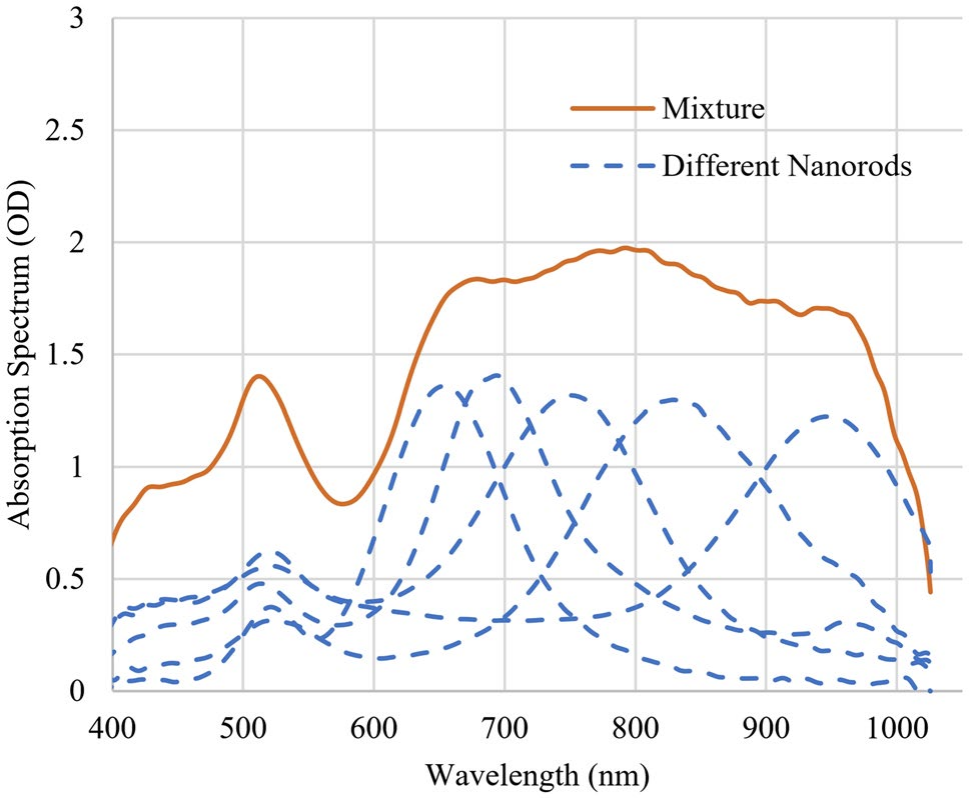
Absorption spectra of different nanorods. The dashed spectra with absorption peaks at 650, 700, 750, 808, and 950 nm are of gold nanorods with the average aspect ratios of 2.4, 2.9, 3.5, 4.1, and 5.5, respectively. The solid line represents the absorption spectrum of the mixture of the five different nanorods with the average aspect ratios from 2.4 to 5.5.

The absorption spectra of the sample solutions in a cuvette with 1-cm length optical path were measured using an OceanView Vis–NIR spectrometer and a tungsten light source. To investigate the homogeneous broadening of a nanorod’s plasmon resonance with the hole burning method, solutions of nanorods with an aspect ratio ranging from 2.4 to 5.5 were used. Resonance peaks ranging from 650 to 950 nm (dashed lines in Fig. 2) were chosen and mixed to create a spectral plateau from 650 to 950 nm as shown in the solid line in Fig. 2. An optical density (OD) equal to 1 corresponds to 10% transmitted light through the samples. This mixture was used to capture interactions of lasers with nanoparticles that have different resonance wavelengths. Non-resonance excitations^43^ do not generate hole structures because nanorods of different sizes and shapes respond uniformly to the light while the resonance plasmon excitation generates a hole in the spectrum^32^. As shown in Fig. 2, the absorption spectra in dashed lines are a wide range of nanorods with different nanorods and the absorption spectrum of their mixture is shown in solid line. A plateau of absorption is obtained in the spectrum of the mixture.

**Figure 3:**
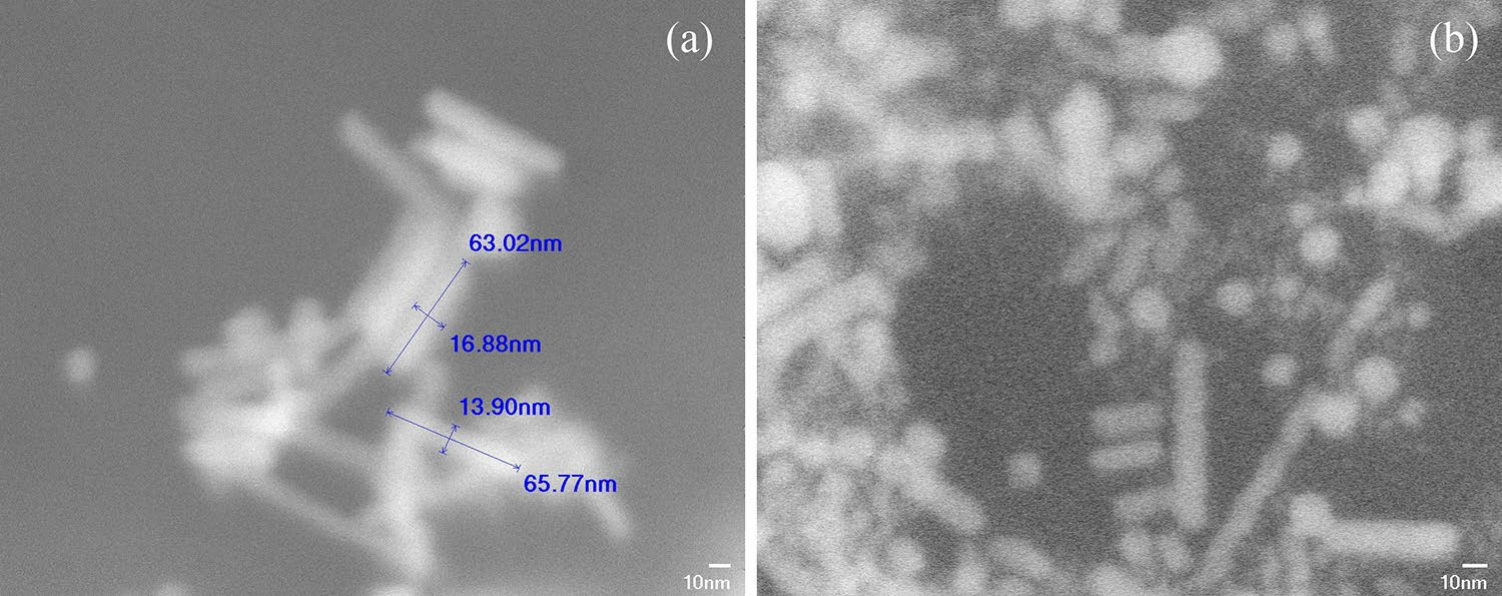
SEM image of an assembly of nanorods. (**a**) before irradiation, nanorods with different sizes and aspect ratio can be seen with length from 30 to 70 nm and width ranging from 10 to 18 nm. (**b**) Nanorods after being irradiated at 10^6^ J/m^2^.

The mixture of gold nanorods with different aspect ratios was placed on a polished silicon wafer and dried in hot air at 50 °C. With a field emission scanning electron microscope (SEM) (JEOL JSM-7401F), SEM images were taken as shown in Fig. 3. The sizes of gold particles range from 10 nm × 70 nm to 15 nm × 25 nm with an aspect ratio of 1.6 to 7, and correspond to longitudinal resonance wavelengths of 550–1000 nm.

**Figure 4:**
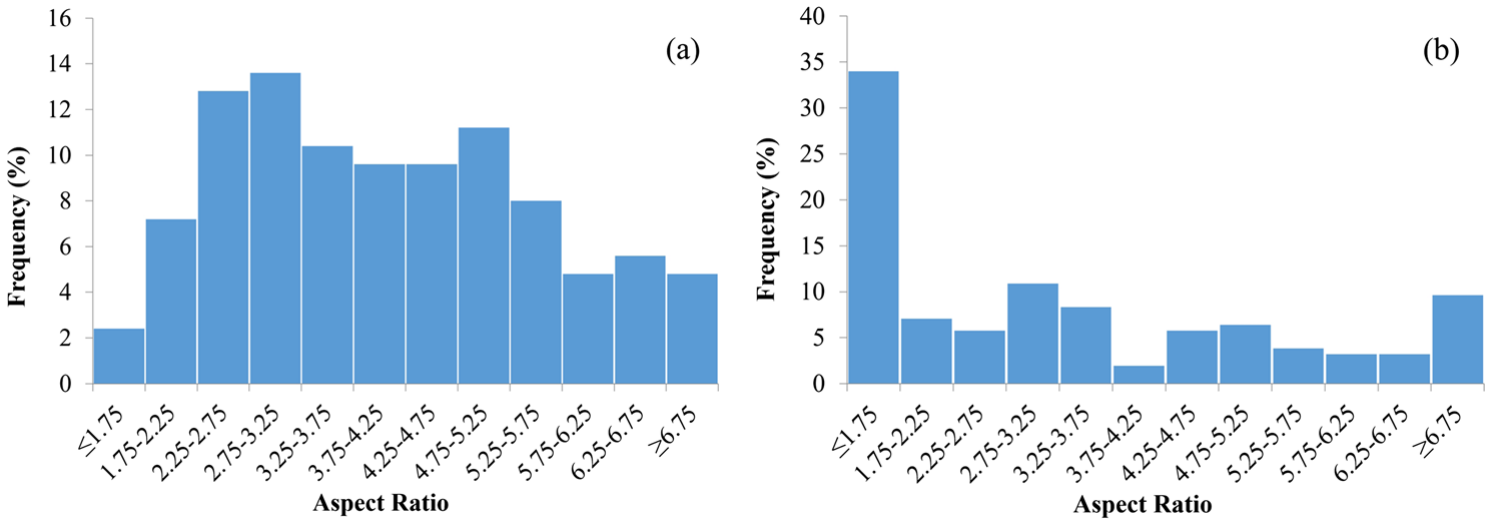
Histogram of aspect ratio before (**a**) and after (**b**) laser irradiation. Nanorods with aspect ratio near 4 reduces in portion, while significant amounts of nanorods with larger aspect ratio (> 6.75) remain.

By comparing SEM images of gold nanorods before and after single pulse scanning, nano spheres with radii ranging from 10 to 30 nm could be found. This result is in agreement with the deformation results proposed by DeSantis et al*.*^32^. Nanorods with longer aspect ratio (> 6.75), could still be seen, while intermediate aspect ratios (3–5) have reduced in portions. The aspect ratio was obtained by measuring the long and short axes of the nanorods in SEM images, and a histogram of its percentage frequency is shown in Fig. 4.

This result coincides with the transmission spectrum that the absorption peak is burned around 800 nm, which corresponds to resonance frequency of gold nanorods of an aspect ratio of 4.1. The formation of nanospheres from the damage of nanorods loses the original longitudinal resonance wavelength around 800 nm, leaving an increased transverse wavelength at 520 nm. The persistence of long aspect ratio nanorods also supports that persistent hole burning method can be used for the study of the homogeneous broadening $\Gamma$ of the resonant plasmon excitation up to the fluence at 10^6^ J/m^2^.

## Results and analysis

**Figure 5:**
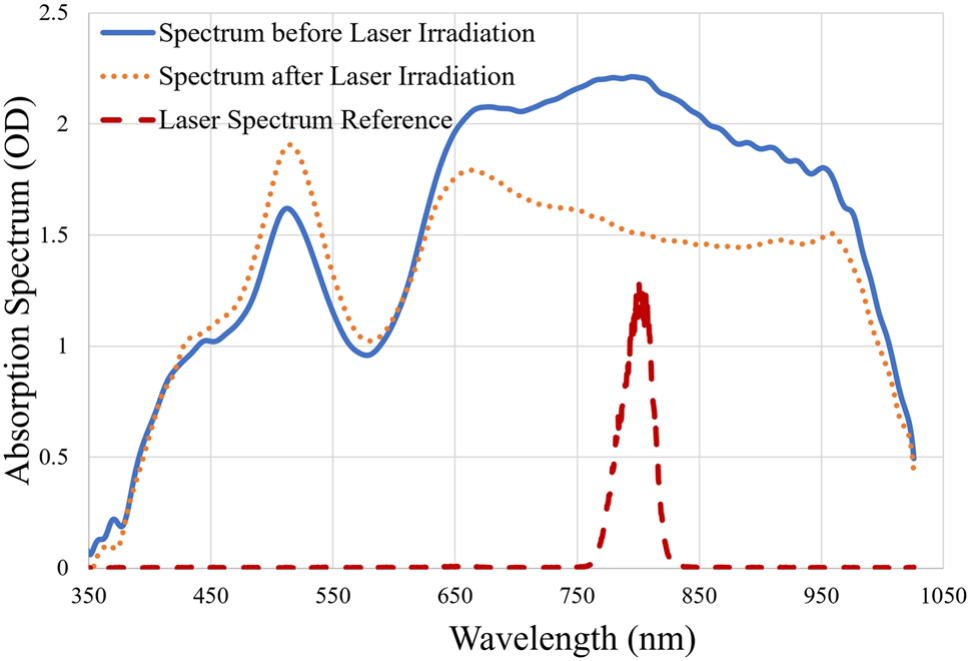
Absorption spectra of nanorod mixture before and after laser irradiation at fluence of 300 J/m^2^.

The absorption spectra of nanorod solutions before and after laser irradiation was taken and an example in Fig. 5 presents the spectra for femtosecond laser fluence at 300 J/m^2^. A distinctive decrease of the optical absorption around 800 nm can be observed, which is much wider than the spectral width of laser as shown in dashed line.

**Figure 6:**
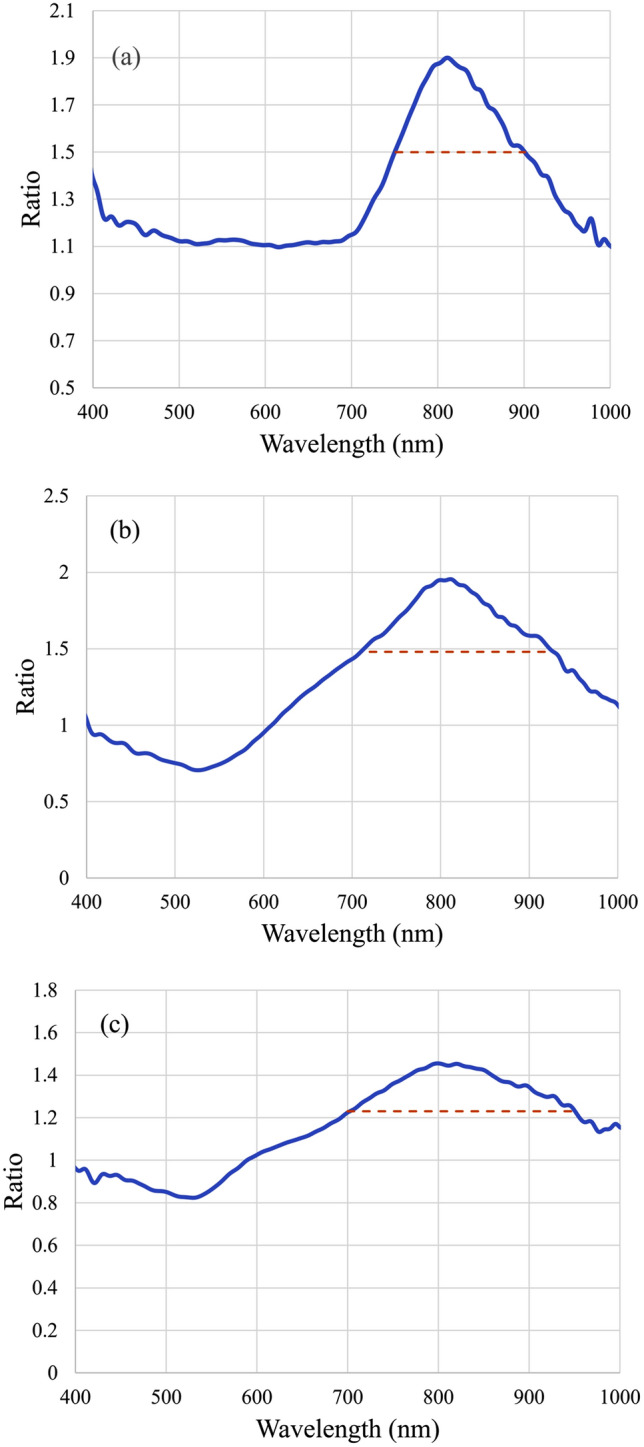
Ratio of absorption spectra before and after irradiating by laser with various fluences. (**a**) For 220 J/m^2^, (**b**) for 1.1 × 10^4^ J/m^2^, and (**c**) for 2.46 × 10^6^ J/m^2^. Widths of spectral holes are labeled with dashed lines.

The spectra were denoised using a Fourier method by filtering out high frequency components. Then the spectra before the irradiation was divided by that after the irradiation to get a ratio spectrum. A decrease in absorption spectrum will result in an increase in the ratio. Typical results are shown in Fig. 6. An obvious peak at about 800 nm could be seen, which is the wavelength of the laser, indicating that the nanorods that resonate with the laser wavelength are destroyed the most. This is very different from the result of DeSantis et al*.*^32^. The hole peak wavelength was very different from the laser wavelength because their nanorods were excited by multiple laser pulses and after a few pulses, the nanorods had already been deformed. A decrease at ~ 520 nm could be seen at higher intensities, corresponding to transverse plasmonic modes of nanospheres and nanorods. It suggests an increase in transverse modes of surface plasmon, which could be found in both nanorods and nanospheres. This result supports the theory of laser induced deformation: nanorods with the 800-nm longitudinal resonance wavelength were melted into spheres with only 520 nm transverse modes.

Figure 6 shows the ratio of absorption spectrum before irradiation to that of after irradiation with laser at various fluences. (a) Shows the ratio of spectra before and after being irradiated at a fluence of 220 J/m^2^. An ablation of spectrum at 800 nm can be seen. (b) Shows the ratio of spectra laser with a fluence of 1.1 × 10^4^ J/m^2^. There is the change at 800 nm, and the generation of nanospheres with transverse resonance wavelength of 520 nm results in a dip. (c) Shows the ratio of spectra before and after laser irradiation at a fluence of 2.46 × 10^6^ J/m^2^. The hole burning effect took place rapidly and peaked at about 800 nm. The hole burning width is measured at the full width at half maximum. If no clear baseline is available, as shown in Fig. 6b,c 1 is used as baseline to calculate half maximum and the resulting widths are shown with dashed lines. Since a femtosecond laser adds extra width from its spectrum, this extra width was canceled by comparing the final results with a convolution of a gaussian distribution and the spectrum of the laser, which lead to a ~ 3 nm decrease. Other broadening effects, including the inter-band transitions^44,45^, temperature^46^ and size^47^ broadening, could be eradicated using a similar convolutional fitting process. These broadening contributions are comparatively small as the spectral hole increases to more than 200 nm, and thus are neglected. The width of the spectral hole increases as intensity increases, implying an increased damping coefficient (i.e., the plasmonic enhancement decreases). Since the number of pulses to create a significant spectral hole varies for different fluences, the experimental conditions varies and thus the absolute ratio is not identical.

**Figure 7:**
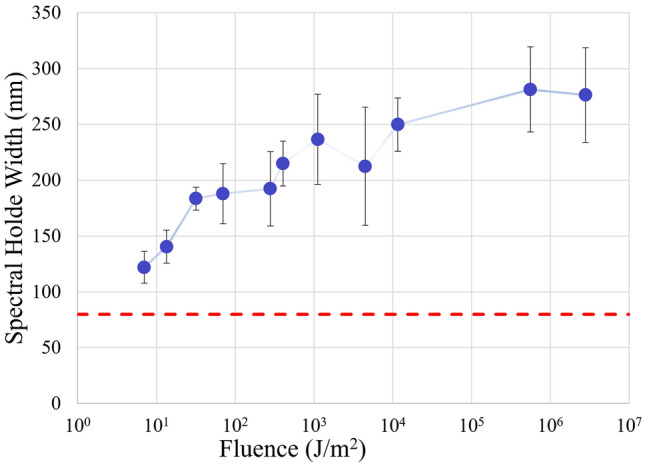
Laser fluence vs. hole burning spectral width in logarithm coordinate. Dots represent experimental data and the lines are simple connections between two nearest data points. Dashed line indicates natural resonance width at a low light intensity.

The results were plotted as a function of laser fluence as shown in Fig. 7. The hole width slowly increased from 149 nm at 11 J/m^2^ to 248 nm at 3 × 10^6^ J/m^2^, compared with low-fluence limit 80 nm, which is labeled by the dashed line. Qiu et al*.*^24^ predicted that when the wavelength of the plasmon resonance of a nanorod increases, its homogeneous broadening also increases. They used a scanning near-field optical microscopy to obtain a homogeneous broadening of about 50 nm for the plasmon resonance at 710 nm. The relationship between the plasmon resonance and its homogeneous broadening predict that the broadening is larger than 60 nm for a plasmon resonance at 800 nm. Weaker nanosecond laser multi-pulse irradiation in Fales et al*.*’s experiment makes a spectral hole with a broadening of about 80 nm at 800 nm in the spectrum of gold nanorods^48^. Therefore, 80 nm was chosen as the reference value of spectral hole burning width at weak light limit.

## Discussion

**Figure 8:**
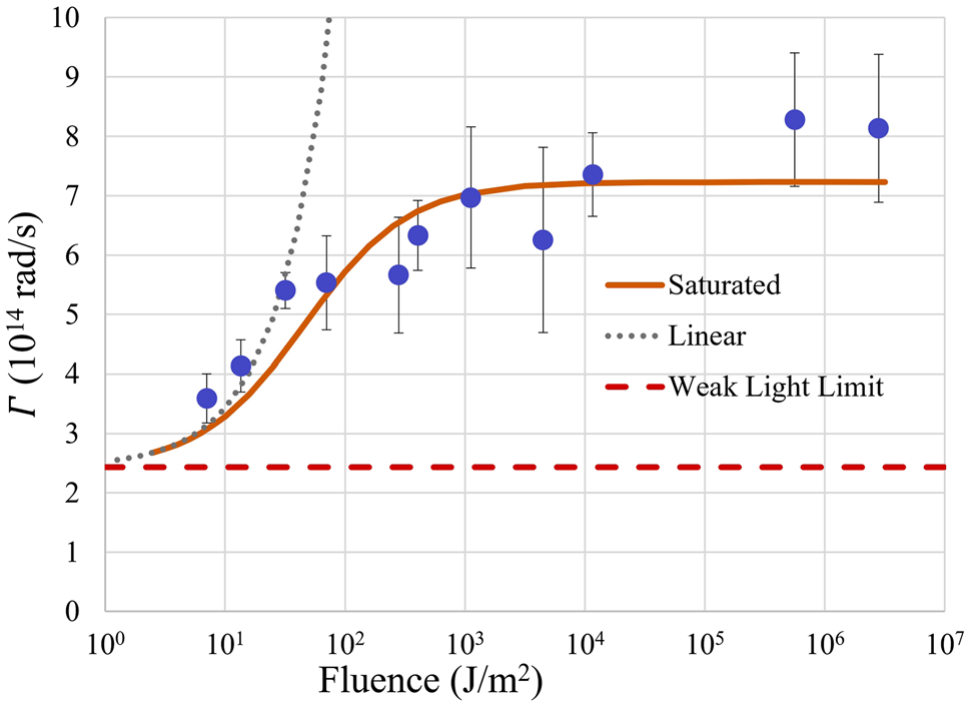
Laser fluence vs. damping coefficient $\Gamma$ in logarithm coordinate. Dots are experimental results; the solid line represents a fitting with a saturation function; the dotted line represents a fitting with a linear function at low fluence and the dashed line represents damping coefficient at weak light limit.

From our experiments, the damping coefficient can be estimated from the hole burning results:

2 $${\text{d}}\omega = \frac{{\left| {{\text{d}}\lambda } \right|2\pi c}}{{\lambda^{2} }}$$

where $d\omega$ is the change in frequency, the damping coefficient is $\Gamma$ in this case, ${\text{d}}\lambda$ is the spectral width from hole burning experiments, $\lambda$ is the wavelength of the spectral hole peak, and c is the speed of light. The damping coefficients obtained at different laser fluences are presented in Fig. 8 with dots.

The interactions between a radiation electric field and free electrons in a metal is well described with the Drude model^49^. The displacement *r* of an electron in the electric field E follows Newton’s second law,

3 $$m_{e} \frac{{d^{2} r}}{{dt^{2} }} = - eE(t) - m_{e} \Gamma \frac{dr}{{dt}}$$

where $m_{e}$ and $- e$ are the mass and electric charge of an electron, respectively, and $\Gamma$ is a damping coefficient that is decided by various scatterings of the electron in its motion. If a harmonic field $E = E_{0} {\text{e}}^{ - i\omega t}$ is applied to Eq. (3), we can obtain the dielectric constant ε(*ω*) of the metal at frequency $\omega$^49^,

4 $$\varepsilon (\omega ) = 1 - \frac{{Ne^{2} }}{{m\varepsilon_{0} }} \cdot \frac{1}{{\omega^{2} + i\Gamma \omega }}$$

where $\varepsilon_{0}$ is the vacuum dielectric constant and $N$ is the electron density in the metal. The optical absorption spectra of a gold nanorod in a medium can be calculated with the dielectric constant $\varepsilon (\omega )$ of gold, the aspect ratio for the nanorod, and the dielectric constant of the medium^50^. Two resonant plasmons modes, i.e., transverse mode and longitudinal mode exist in the absorption spectrum. The longitudinal mode largely depends on the aspect ratio for the nanorod and the dielectric constant of the medium. The line spectral width of the plasmon resonant peaks is determined by $\Gamma$, which is originated from the electron scattering with nanorod’s surface $\Gamma_{e - surface}$, the scattering with phonons $\Gamma_{e - phonon}$, and the scattering of the electron with another electron $\Gamma_{e - e}$. We have observed that the $\Gamma$ increases when the single pulse fluence increases. $\Gamma_{e - surface}$ cannot explain the change of $\Gamma$ since this only relates to the shape and size of the nanorods. $\Gamma_{e - phonon}$ cannot explain the increase, since observed $\Gamma$ is less than 100 femtosecond, which is shorter than the time it takes for absorbed energy to be transferred to the phonons from hot electrons^37^. For $\Gamma_{e - e}$, the electron–electron scattering rate was studied as early as in 1958, by Gurzhi^51^, and it was calculated in detail later in 1973 by Lawrence^52^. They used Born approximation and the Thomas–Fermi screening of Coulomb interaction to describe frequency and temperature dependence of the collision process among electrons^51^,

5 $$\Gamma_{e - e} = \frac{{\pi^{3} }}{{12hE_{F} }}\left[ {\left( {k_{B} T} \right)^{2} + \left( {\frac{h\omega }{{2\pi }}} \right)^{2} } \right]$$

where $h$ is the Planck’s constant, $k_{B}$ is the Boltzmann’s constant, T is the temperature, and $E_{F}$ is the Fermi energy, for gold $E_{F} = 5.5 \;eV$. The calculation of the $T^{2}$ and $\omega^{2}$ dependences are based on the assumption that electrons before and after scattering are close to the Fermi surface. By using the cross section of a gold nanorod^41^ and the laser fluence, we estimate that when the fluence is larger than 100 J/m^2^, in the nanorod, the photon number is larger than the gold atoms. In such a high excitation where most of the electrons are excited, $E_{F}$ and T are not well defined since the system has not reached thermal equilibrium before scattering completes, and thus Eq. (5) cannot be used. At excitation, before the electron movement’s dephasing, most of the electrons oscillate synchronously and the scattering event per electron among them should approach zero. Therefore, electron–electron scattering cannot explain the $\Gamma$ increase with fluence increase, either.

At such a high excitation, electrons are undergoing plasmonic oscillation together, and the photon number is so large that we cannot neglect the plasmon–photon interaction^35^. Plasmon and photon are both bosons, so when their numbers are large, the interaction becomes significant. Following Finazzi and Ciccacci’s derivation^35^, with their the plasmon–photon interaction $H_{pl - ph}$, we consider the process: a plasmon in $\upomega$ state with the occupation number of $n_{\upomega}$ decaying into a photon in a state with wave vector ***k*** and polarization u_α_ and occupation number of ${\text{n}}_{{{\varvec{k}},\alpha }}$. The probability per unit time for a plasmon to decay into a photon with wave vector ***k*** and polarization *u*_***α***_ is given by the Fermi golden rule^35^:

6 $$\begin{aligned} T_{\upomega,{{\varvec{k}}},a} & = \frac{2\pi }{\hbar }\left| {n_{\upomega} - 1,n_{{{\varvec{k}}},\alpha } + 1\left| {H_{pl - ph} } \right|n_{\upomega} ,n_{{{\varvec{k}}},\alpha } } \right|^{2} \delta \left( {\hbar \upomega_{\upomega} - \hbar \upomega_{k} } \right) \\ & = \frac{{V_{p} }}{{2\pi c^{3} }}\upomega_{k }^{4} n_{\upomega} \left( {n_{{{\varvec{k}}},\alpha } + 1} \right) \\ \end{aligned}$$

where c is light speed, $\omega_{w} \;{\text{and}} \;\omega_{{\varvec{k}}}$ are the angular frequency of the plasmon and photon, and $V_{p}$ is the volume of a box containing the nanorod and the radiation. Because of this plasmon–photon interaction, the plasmon lifetime τ_w_ is

$$\tau_{w} = \frac{{n_{w} }}{{T_{w,{{\varvec{k}}},\alpha } }}$$

And the damping coefficient^35^ is

7 $$\Gamma_{\omega} = \frac{1}{{\tau_{\omega} }} = \frac{{V_{p} \omega_{k}^{4} }}{{2\pi c^{3} }}\left( {n_{{{\varvec{k}}},\alpha } + 1} \right)$$

The factor $(n_{k,\alpha } + 1)$ in Eq. (7) shows that $\Gamma$ increases as the light intensity increases. Subsequently, we introduce a simple linear relation where the $n_{k,\alpha }$ is proportional to the laser fluence $I$:

8 $$\Gamma = \Gamma_{0} + \alpha I$$

where ${\Gamma }_{0}$ is damping coefficient when light intensity is very weak, $\alpha$ is a linear proportional coefficient. As shown in Fig. 8, in logarithm coordinate, the linear model is represented by an exponential curve, as shown by a dotted line. At a high intensity, the points deviate significantly from this trend since at a high fluence, this linear intensity dependence assumption from Eq. (8) fails and the rising trend slows. The photon occupation number $n_{k,\alpha }$ in Eq. (7) is not a simply linear function of laser fluence *I*. The photon occupation number $n_{k,\alpha }$ must be a monotonically increasing function of fluence *I*. To fit the experimental results, we may use:

9 $$\Gamma = \Gamma_{0} + \frac{\alpha I}{{1 + \frac{I}{{I_{s} }}}}$$

where $I_{s}$ and $\alpha$ are the fitting parameters, $I_{s}$ is saturation intensity, and $\alpha$ is the same proportionality constant in Eq. (8), while $\Gamma_{0}$ is the low energy limit and is a constant that is 2.34 × 10^14^ rad/s. The fitted values are $I_{s} = 46\;{\text{J/m}}^{2}$ and $\alpha = 1.04 \times 10^{13} \; {\text{rad}} \cdot {\text{m}}^{2} \;{\text{s}}^{ - 1} \;{\text{J}}^{ - 1}$. The fitted curve is presented as the solid line. The trend matches the general behavior that $\Gamma$ is mostly linear at low fluences, exponential in logarithm coordinates, and then is saturated to a higher value. This saturation could be explained by simultaneous emission: that at very extreme intensity, plasmon interact with light and re-emit photons faster, and the duration of the pulse is longer than the lifetime of the plasmon, so exceeding energy would not contribute to photon occupation number fully.

## Conclusion

A theory for plasmonic behavior based on the Drude model was built to relate spectral hole width with damping coefficient $\Gamma$. The persistent hole burning in the longitudinal mode band of gold nanorods irradiated by a single laser pulse shows that $\Gamma$ is tripled to about 7 × 10^14^ rad/s at an average fluence of 10^6^ J/m^2^, compared to 2 × 10^14^ rad/s at weak light intensity. The results indicate that the damping coefficient increases sub-linearly with laser fluence. Using plasmon–photon interaction^35^, the increase of the damping coefficient was explained by the boson property of photons being ineligible when the photon number or light intensity is high. The experimental results show that the surface plasmonic effect holds at extreme femtosecond laser fluence, remaining 10% plasmonic enhancement of light at the fluence of about 10^6^ J/m^2^.
